# The impact of experience on recurrence rates after biopsy punch excision for pilonidal disease

**DOI:** 10.1111/codi.16126

**Published:** 2022-05-15

**Authors:** Luigi Basso, Renato Pietroletti, Alessandro Micarelli, Agreta Bicaj, Umberto Costi, Daniele Crocetti, Giuseppe D'Ermo, Gaetano Gallo

**Affiliations:** ^1^ ‘Pietro Valdoni’ Department of Surgery, Faculty of Medicine and Dentistry, Policlinico ‘Umberto I’ ‘Sapienza’ University of Rome Rome Italy; ^2^ Surgical Coloproctology, ‘Val Vibrata’ Hospital L'Aquila University TE Italy; ^3^ ITER Center for Balance and Rehabilitation Research (ICBRR) Rome Italy; ^4^ Eurac Research, Institute of Mountain Emergency Medicine Bolzano Italy; ^5^ Unit of General Surgery and Surgical Oncology, Department of Medicine, Surgery and Neurosciences University of Siena Siena Italy

**Keywords:** Bascom, biopsy, cyst, Gips, minimally invasive surgery, pilonidal, punch, sinus

## Abstract

**Aim:**

We present the outcomes and the recurrences of 848 patients with pilonidal disease (PD) treated by biopsy punch excision (BPE) and we weigh our results against progressively obtained operative experience. BPE is a modified ‘merged’ version of both the Bascom ‘pit picking’ procedure and the Gips procedure. It employs biopsy punches of different calibre, depending on whether treatment is in the natal cleft (calibre as small as possible) or lateral (larger calibre punches or even small incision). Sometimes this procedure is referred to as the Bascom–Gips procedure.

**Methods:**

In all, 848 consecutive patients with PD were treated from January 2011 until December 2016 (sex 622 [73.4%] men and 226 [26.6%] women; median age 26.2 years, mean age 24.6 ± 28.99 [range 14–55] years, men 25.1 years, women 24.8 years). Of these 848 patients, 287 were operated in 2011–2012, 301 in 2013–2014 and 260 in 2015–2016. The recurrence rates were recorded 12, 24 and 60 months after surgery both cumulatively and by examining the outcomes of the three biennia individually (years of treatment 2011–2012 or group A, 2013–2014 or group B, 2015–2016 or group C).

**Results:**

The mean operating time was 34 ± 24.45 min. Postoperative complications included early (<24 h; *n* = 22 or 2.6%) and delayed (>24 h; *n* = 26 or 3.1%) postoperative bleeding. Postoperative fluid collections (<2 weeks) occurred in 83/848 patients (9.8%) and included haematoma (*n* = 25) and seroma (*n* = 58). Full recovery was obtained after a mean of 21 ± 12.72 days and work/school/university activities were resumed after a mean of 4 ± 12.02 days. Twelve‐, 24‐ and 60‐month follow‐ups were possible in 725 (85.5%), 682 (80.4%) and 595 (70.2%) patients out of 848. An overall significant (*ꭓ* ^2^ = 16.87, *P* = 0.0002) difference was found in the recurrence rates: 59 recurrences/725 patients (or 8.1%) after 1 year, 89 recurrences/682 patients (or 13.0%) after 2 years and 98 recurrences/595 (or 16.4%) after 5 years. However, when subgrouping patients in three 24‐month subsets, the recurrence rates showed a steady and progressive decrease in the three biennia 2011–2012 (group A), 2013–2014 (group B) and 2015–2016 (group C) at 12‐, 48‐ and 60‐month follow‐ups. Recurrences after 12 months were 29/225 (12.9%), 19/285 (6.7%) and 11/215 (5.1%) (*ꭓ* ^2^ = 8.53, *P* = 0.014) in groups A, B and C respectively; after 24 months, 36/226 (15.9%), 31/242 (12.8%) and 22/214 (10.2%) (*ꭓ* ^2^ = 2.38, *P* = 0.30 N.S.) in groups A, B and C respectively; after 60 months, 38/194 (19.5%), 36/215 (16.7%) and 24/186 (12.9%) (*ꭓ* ^2^ = 2.23, *P* = 0.32) in groups A, B and C respectively.

**Conclusions:**

BPE is an effective, disease‐targeted, minimally invasive and inexpensive way to treat PD. Its results are influenced by the experience of the team involved, especially regarding early recurrences/failure of surgery. At least 5‐year follow‐ups are needed to ascertain the outcome of surgery for PD.

**What does this paper add to the literature?**

Our study highlights the impact of surgeons' experience on both success and recurrences after punch biopsy cystectomy/fistulectomy. The good results obtained suggest that the technique should be an arrow in every proctologist's bow. An appropriate and validated definition of ‘recurrence’ and a follow‐up >5 years are necessary.

## INTRODUCTION

In the early 1980s Bascom showed that PD originates from hair follicles, hence giving solid scientific grounds to his minimally invasive surgery (MIS) approach [1, 2], stating that ‘wide excision of blocks of fat down to periosteum, an outmoded treatment, now seems equivalent to treating a pimple on the chin by cutting off the patient's head!’ [3]. In 2008, Gips et al. [4] slightly modified Bascom's original MIS technique by employing trephines and disposable biopsy punches to excise only the diseased PD tissue. Recently, other MIS techniques have been developed, employing instruments such as fistuloscopes or hysteroscopes [5, 6]. We perform biopsy punch excision (BPE), which is a modified ‘merged’ version of Bascom's ‘pit picking’ procedure and the Gips trephines procedure and employs biopsy punches often of different calibre, depending on whether treatment is in the natal cleft (calibre as small as possible) or lateral (larger calibre punches or even a small incision is performed). Sometimes BPE is referred to as the Bascom–Gips procedure. We present the outcome of 848 PD patients operated from 2011 until 2016 by means of BPE and weigh PD recurrences against the progressively achieved experience with this technique. The hypothesis is that removal only of diseased tissue suffices to treat PD (‘see and treat’) and that this approach becomes particularly effective with experience.

## MATERIALS AND METHODS

This was a retrospective single‐centre study and is reported according to the Strengthening the Reporting of Observational Studies in Epidemiology (STROBE, see Appendix 1) statement for cohort studies [7].

We first adopted BPE in 2010. Subsequently, from January 2011 to September 2016, 848 consecutive patients with PD were treated employing BPE. Of these, 148 (17.5%) had received previous drainage of PD abscess, while 89 (10.5%) had received previous surgery other than simple drainage (time range from previous surgery 1–12 years). Exclusion criteria included more than two recurrences, extensive disease, recurrent or nonhealing large open wounds, mostly after wide excision operations performed elsewhere, sacrococcygeal area severely deformed, coagulation disorders, and patient's refusal to consent for the MIS procedure. Diagnosis was clinical and later confirmed by pathology. The procedure was always performed as a day‐case by the same surgeon (BL) assisted by the same team at ‘Pietro Valdoni’ Department of Surgery, Policlinico ‘Umberto I’, ‘Sapienza’ University of Rome, Faculty of Medicine and Dentistry, Rome, and ‘Ars Medica’ Private Hospital, Rome, Italy. The study population (*n* = 848) included *n* = 622 (73.3%) men and *n* = 226 (26.6%) women (mean age 24.6 ± 28.99). Of these 848 patients, 287 were operated in 2011–2012 (group A), 301 in 2013–2014 (group B) and 260 in 2015–2016 (group C). During the first few weeks after surgery, our patients were always checked once or more, if needed, until complete wound healing was obtained. This typically occurred in just a few weeks, except in those progressing towards the status of ‘non‐healing wound(s)/persistence of PD/early recurrence' (<1 year) (see below, Discussion), which required longer clinical attention. In this early postoperative period, all our patients were examined directly by us or, if they were from far away locations, at their general practitioner’s surgery. In the following months and years, our patients were considered to have a recurrence if they required reoperation or reported local pain, discharge or intermittent swelling, as assessed, whenever possible, by means of direct clinical examination or, if this was not possible, by interviews via telephone or email or WhatsApp, or as reported to us by general practitioners or by another doctor or attending nurse. Recurrent PD was recorded at 12, 24 and 60 months after surgery, and the figures were analysed both cumulatively and by examining the outcomes in each of the three biennia taken into consideration.

### Surgical technique

**FIGURE 1:**
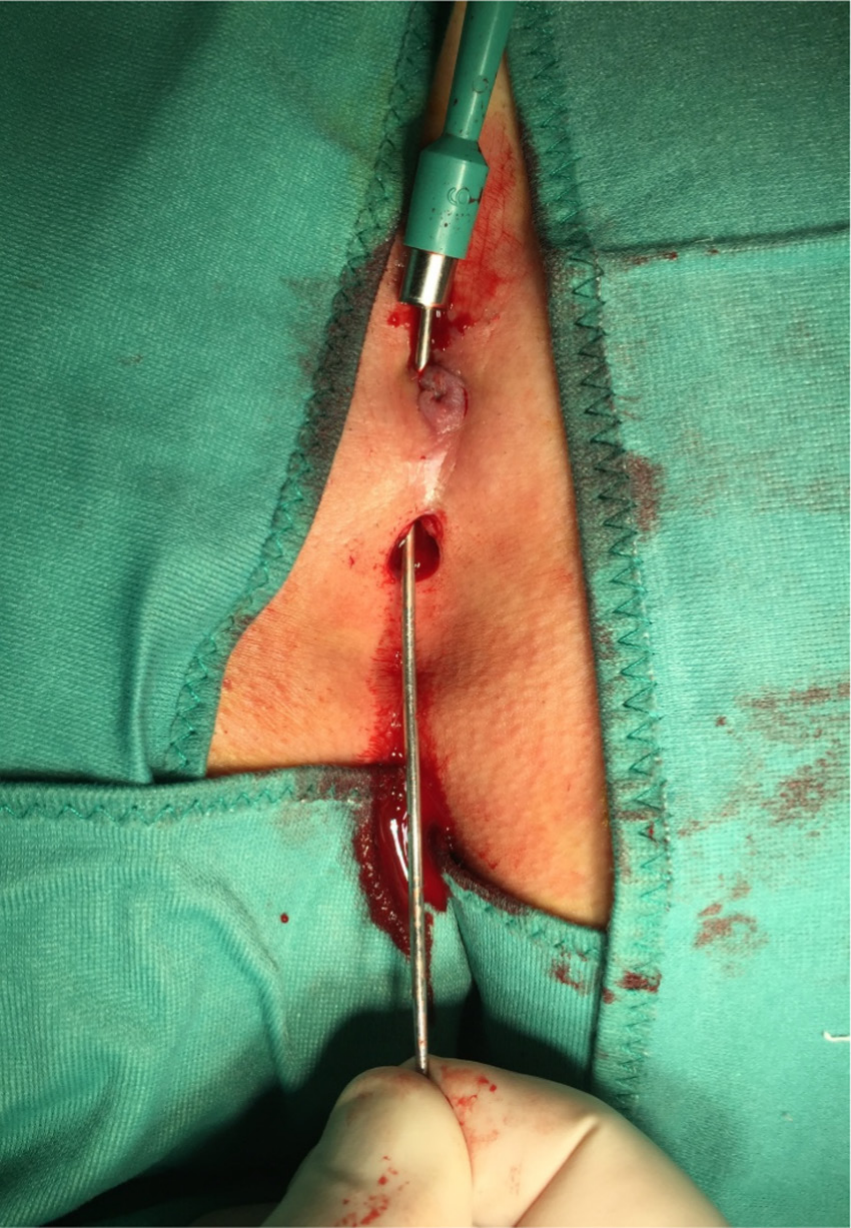
Coring out is performed by the biopsy punch, under guidance of the surgical probe

**FIGURE 2:**
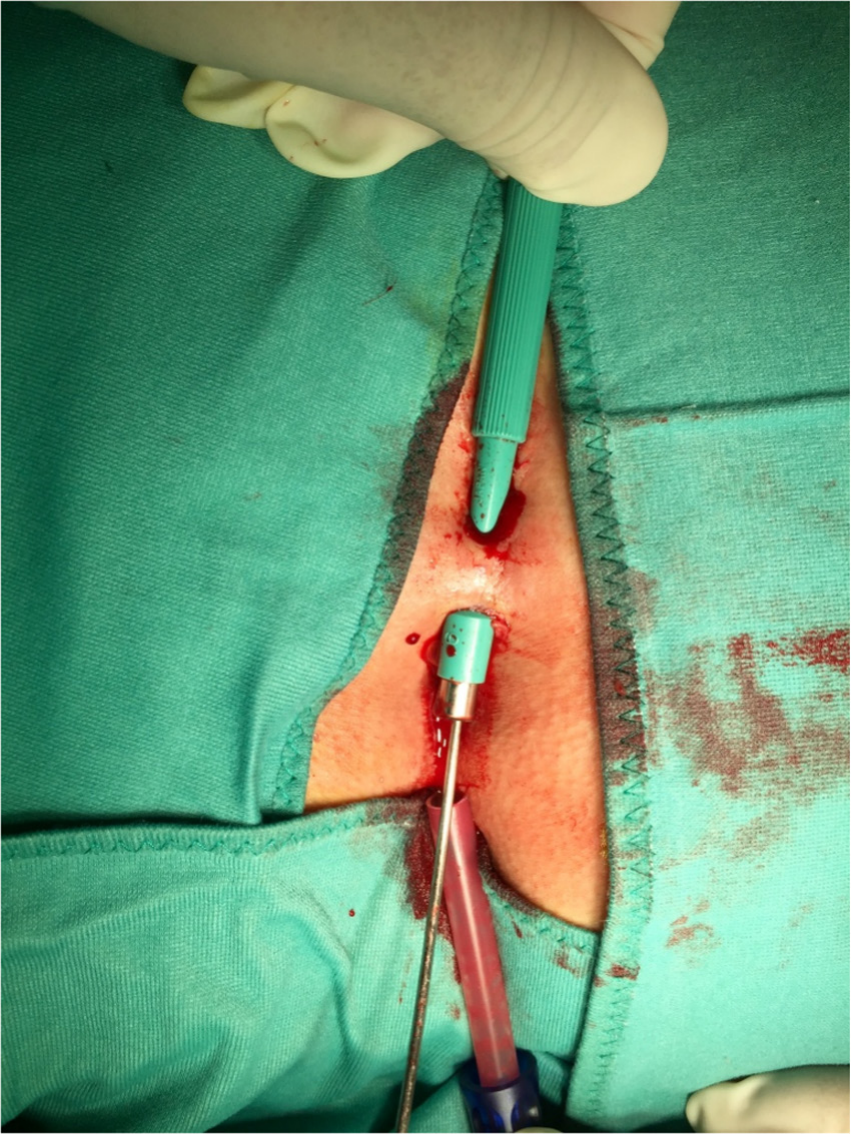
Coring out by the punch biopsy is completed

**FIGURE 3:**
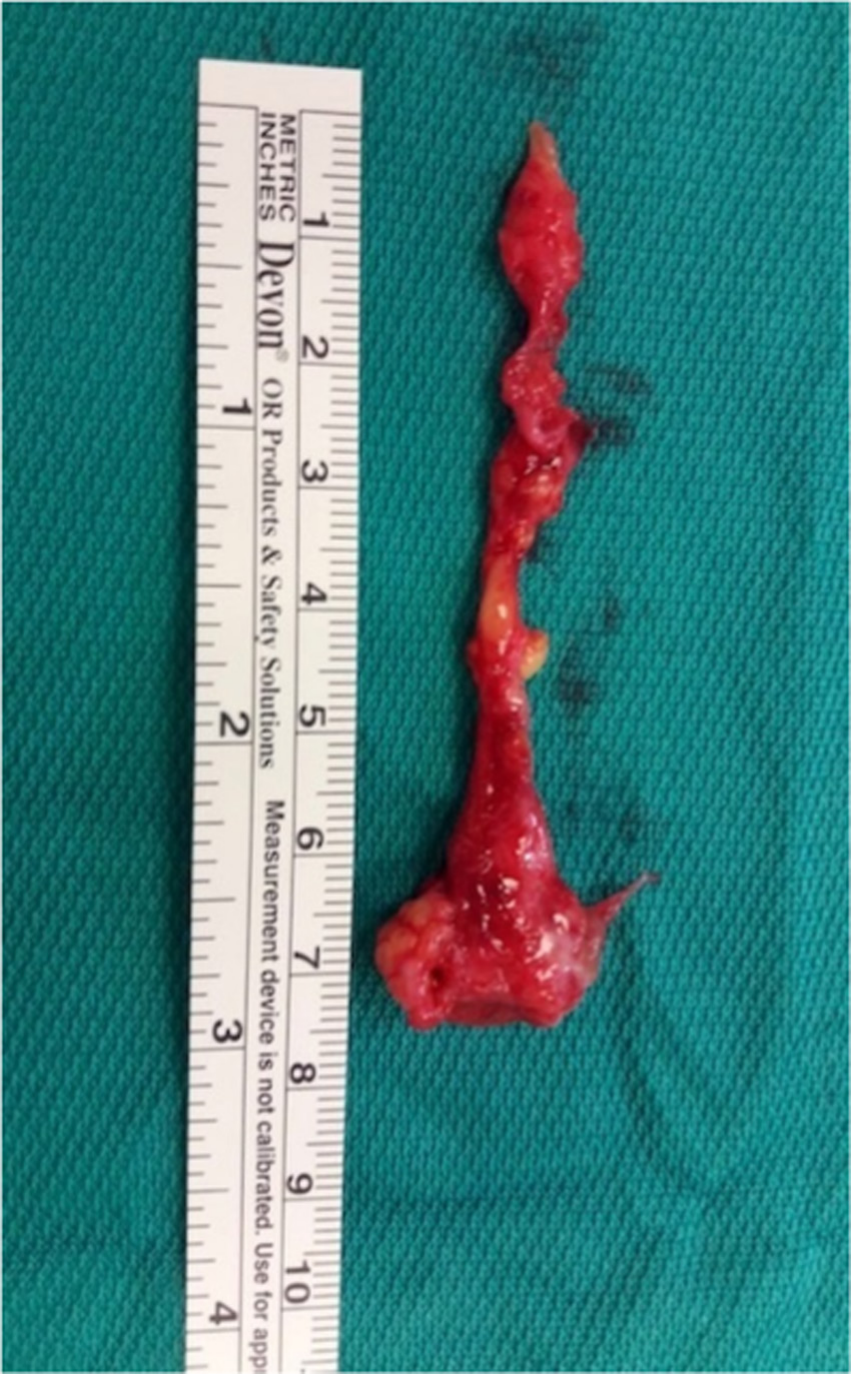
The removed specimen includes pilonidal cyst and fistula

Carbocaine (2%) with 1 ml of bicarbonate is injected for local anaesthesia (mean volume 8, range 5–15 ml). Mild sedation (Hypnovel^®^ intravenous [i.v.] 5 mg, Roche) or deep sedation (Propofol i.v. 20 mg, B. Braun) or no sedation at all is given by the anaesthetist, according to the individual PD and to the patient's compliance and personal wishes. Occasionally, i.v. pain relief is also given intra‐operatively (Fentanest^®^ 100 μg, Pfizer). All subjects are operated in the prone position and, after complete shaving, the buttocks are thoroughly examined with the help of a surgical probe and palpated, to look for further cyst(s) and sinus track(s), sometimes only detected under stretching (what we call the ‘violin string’ effect). Biopsy punches (Kai Medical, Japan) are used with a calibre of 3.0, 3.5, 4, 5, 6 or 8 mm, according to the size of the pit(s) and the extent of the disease. Often, two different sizes are used in the same patient, employing the larger calibres for lateral disease and approach and the smaller calibres for PD in the natal cleft. Coring out is first performed by the biopsy punch, possibly under guidance of the surgical probe (Figures 1 and 2). Following this, a small blade scalpel (size <15), small scissors, Kocher's forceps, a Volkmann spoon and a disposable dermal curette (Kai Medical) are used to thoroughly remove any remaining debris, residual fibrosclerotic tissue and abscess walls, possibly staying ‘out of the ditch’ and therefore as lateral as possible [3]. At the end, after generous washing out with normal saline, the small central wounds are sutured with loosely applied Vicryl^®^ Rapide 3/0 or 2/0 (Ethicon) stitches, while the lateral wounds are left open for drainage. The cyst(s) and debris are sent to the pathologist for microscopic evaluation (Figure 3). Openings larger than 8 mm (maximum calibre of disposable biopsy punches) are handled by enlarging the wound by means of a size 15 mm scalpel, removing only the necessary diseased tissue. If the PD does not present any lateral involvement but only midline pits, these are first treated and removed by means of biopsy punches of the smallest size to achieve removal, and the resulting small central wounds are sutured, again with loosely applied Vicryl^®^ Rapide 3/0 or 2/0 (Ethicon). Large calibre (e.g., 8 or 6 mm) biopsy punches are employed, hence creating lateral wound(s) on one or both sides, to perform optimal debridement of diseased tissue from the lateral aspect towards the midline (‘stay out of the ditch’) and to favour postoperative wound irrigation and drainage of fluid collections towards the outside.

### Postoperative care

A compressive dressing (Tensoplast®, BSN Medical) is applied and the patients are encouraged to lie supine in bed, applying pressure on their dressing for the first 24 h. After removal of the compressive dressing, an occlusive plaster is applied, and the patients are encouraged to keep changing these plasters twice daily at home for the first 2 weeks, following showers with running tap water, irrigating the open wounds for at least 5 min each time. Electrical water flosser may also be employed to irrigate the open wounds. Postoperatively, oral paracetamol (1 g tablet) is prescribed and taken if needed (8 hourly for 3 days).

### Data handling and statistical analysis

Descriptive data were calculated as mean ± SD. The *χ* ^2^ test was carried out to define associations between categorical factors and groups. To assess whether data for independent samples were of Gaussian distribution, D′Agostino's *K* ^2^ normality and Levene's homoscedasticity tests were applied (where the null hypothesis is that the data are normally and homogeneously distributed). A significant cut‐off level (*α*) was set at a *P* value of 0.05 (Statistica 7 package for Windows) [8].

## RESULTS

The mean operating time was 34 min (median 35, range 19–55). Postoperative complications included early (<24 h; *n* = 22 or 2.6%) and delayed (>24 h; *n* = 26 or 3.1%) postoperative bleeding. Early postoperative bleeding was always successfully treated by lying the patient supine on a hard surface for 60 min or so [9]. Postoperative pain, if present, was always controlled by paracetamol (1 g tablet, 8 hourly) for the first 3 days, except in eight cases (0.9%) where it lasted for more than 1 week. Postoperative fluid collections (<2 weeks) occurred in 83/848 patients (9.8%) and included haematoma (*n* = 25) and seroma (*n* = 58). Postoperative paraesthesia at or near the operation site was observed in two cases (0.2%), while wound infection was recorded in five (0.6%) patients. Full recovery was obtained in 21 ± 12.72 days. Daily activities were resumed in 4 ± 12.02 days. At 12, 24 and 60 months postoperative full recovery was obtained in 21 days (mean), 18 (median 18), 11–29 (range). Twelve‐, 24‐and 60‐month postoperative follow‐ups were possible in 725 (85.5%, 12 months), 682 (80.4%, 24 months) and 595 (70.2%, 60 months) out of 848 patients.

**FIGURE 4:**
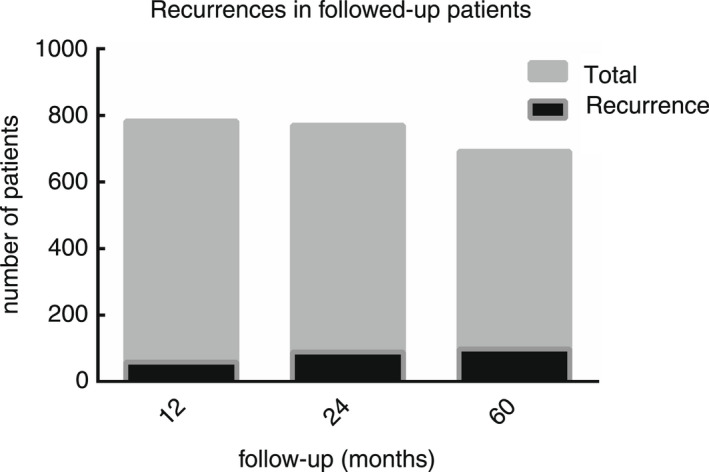
Cumulative recurrences in followed up patients at 12, 24 and 60 months after surgery

**FIGURE 5:**
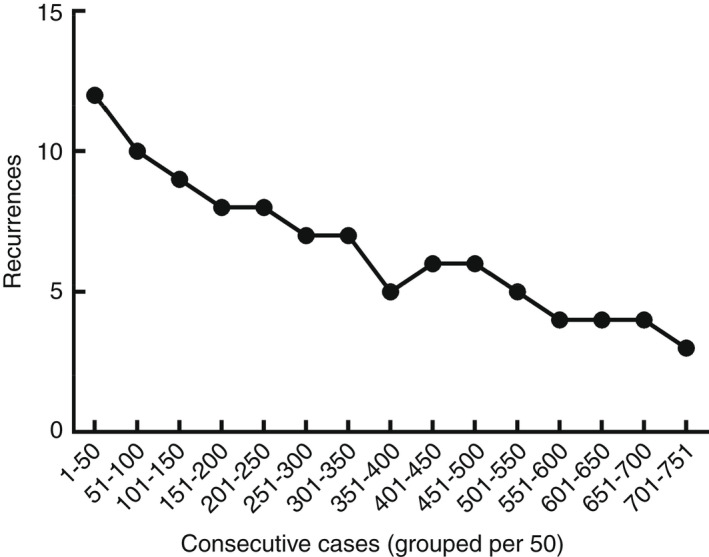
Number of recurrences (on *y* axis) plotted against the cumulative cases (on *x* axis) grouped per 50

An overall significant difference (*ꭓ* ^2^ = 16.87, *P* = 0.0002) was found in the recurrence rate along the follow‐up points—the longer the follow‐up, the higher the recurrence rate (Figure 4). However, when subgrouping patients in three 24‐month subsets, the recurrence rates showed a steady and progressive decrease in the three 2011–2012 (group A), 2013–2014 (group B) and 2015–2016 (group C) biennia at 12‐, 48‐ and 60‐month follow‐ups (Figure 5 and Table 1). Figure 5 depicts the trend of recurrences with respect to consecutive cases.

**TABLE 1:** Recurrences in followed up patients

	Recurrences	Total	*ꭓ* ^2^
12 months	59 (8.1%)	725	17.65
24 months	89 (13%)	682	
60 months	98 (16.4%)	595	
Subsets	**12 months**		
2011–2012	29 (12.8%)	225	8.53
2013–2014	19 (6.6%)	285	
2015–2016	11 (5.1%)	215	
	**24 months**		
2011–2012	36 (15.9%)	226	2.38
2013–2014	31 (12.8%)	242	
2015–2016	22 (10.2%)	214	
	**60 months**		
2011–2012	38 (19.5%)	194	2.23
2013–2014	36 (16.7%)	215	
2015–2016	24 (12.9%)	186	

Number of recurrences in patients undergoing 12‐, 24‐ and 60‐month follow‐up.

## DISCUSSION

In 2008 [4], Gips et al. reported the outcome of MIS for PD in 1358 patients, based on the Bascom principles and approach [1, 2, 3] and employing trephines, replaceable by widely available and low‐priced disposable biopsy punches (around €3.00 or US$3.50 each, exchange rate in February 2022). Both trephines and biopsy punches make Bascom's rice‐grain‐shaped scalpel incisions quicker and easier to perform with sizes ranging from 3.0 to 8 mm. Through the punch wounds, careful excision of fistulas, their pits, abscess cavities and fibrosclerotic tissue is possible (Figure 3). Following the employment of the biopsy punch, a small blade scalpel, small scissors, Kocher's forceps and a Volkmann spoon should also be employed through the ‘keyhole’ created by the punch and sometimes enlarged for further removal of diseased tissue. Satisfactory excisions of PD can therefore be performed, especially from the lateral aspects, always keeping in mind the Bascom ‘stay out of the ditch’ mantra. In fact, lateral wounds, even if wide, heal very easily compared to midline wounds [3]. BPE is not technically difficult; nevertheless it requires attention, experience and understanding of the individual PD and the possibility of a recurrence or of a pending failure. Regarding the term ‘recurrence’ after PD surgery, we should distinguish between (1) *recurrence*—true recurrence, occurs >1 year after surgery in the same area, after illusory ‘healing’; (2) *new localization* of PD, in a non‐operated, previously unaffected area of the natal cleft (MIS only treats small areas and PD might arise de novo in an area different from first site); (3) *failure—*non‐healing wound(s)/persistence of PD/early recurrence (<1 year). In BPE all three instances may occur, and in this study we have labelled all three circumstances as ‘recurrences’.

An overall significant (*ꭓ* ^2^ = 16.87, *P* = 0.0002) difference was found in the recurrence rate along the follow‐up points: 59/725 patients (or 8.1%) after 12 months, 89/682 patients (or 13.0%) after 2 years and 98/595 (or 16.4%) after 5 years. This confirms the importance of long enough follow‐up, ideally for at least 5 years [10]. Furthermore, when subgrouping patients operated in three biennia, the recurrence rates showed a steady and progressive decrease after 12, 48 and 60 months from operation parallel to growth of experience of the surgical team, comparing patients operated in 2011–2012 versus 2013–2014 versus 2015–2016. Hence, albeit statistically significant differences were only detected when comparing early recurrences or ‘failures’ (<12 months) of the three biennia, also follow‐ups after 24 and 60 months showed improving trends, with decreasing recurrences. These trends did not reach statistical significance, yet 5‐year recurrences fell from 19.5% (in group A or first biennium 2011–2012) to 12.9% (in group C or third biennium 2015–2016), which confirms the importance of surgical ‘experience’ in BPE. ‘Learning curves’ are the visual representation of acquired experience, representing the rate of learning alongside repeated experiences or over time. Learning curves were first described in snails [11] and in the aircraft industry [12], and their importance has later been stressed in conventional, laparoscopic [13] and hi‐tech surgery [14, 15]. Our research confirms the importance of experience, especially in decreasing the rate of ‘failed’ surgery, even in BPE for PD.

Full assessment of PD can be tricky; therefore other MIS techniques have been developed [5, 6, 16, 17] which entail the use of a fistuloscope/paediatric hysteroscope, an obturator, a monopolar electrode (to be changed every few sessions), a brush and endoscopic forceps, all the equipment costing around €8000 or US$9400. These endoscopic procedures are based on the same sound approach; however, equally good results may be achieved with inexpensive instruments as long as these are handled by specially dedicated surgeons.

This study has some limitations. Although the data were collected prospectively, data analysis was only performed retrospectively. Besides, the lack of a control group may reduce the strength and impact of our results.

## CONCLUSIONS

BPE is an effective, disease‐targeted and inexpensive MIS way to treat PD, simple to perform but better handled by surgeons with experience in this technique, especially when early ‘recurrences’ (or ‘failures’) are considered. Its low cost, low recurrence rate, short operating time and rapid postoperative recovery all recommend BPE. Finally, after surgery for PD, at least 5 years of follow‐up are needed to determine the surgical outcome.

## CONFLICT OF INTEREST

All authors declare no personal conflict of interest.

## AUTHOR CONTRIBUTIONS

LB & GG contributed equally to this work: Substantial contributions to the conception and design of the work; acquisition, analysis, and interpretation of data for the work. Drafting the work and revising it critically for important intellectual content. Final approval of the version to be published. Agreement to be accountable for all aspects of the work in ensuring that questions related to the accuracy and integrity of any part of the work are appropriately investigated and resolved. AM & RP contributed equally to this work: Substantial contributions to the conception and design of the work; acquisition, analysis, and interpretation of data for the work. Agreement to be accountable for all aspects of the work in ensuring that questions related to the accuracy and integrity of any part of the work are appropriately investigated and resolved. AB, UC, DC & GD contributed equally to this work: substantial contributions to the acquisition of data for the work. Final approval of the version to be published.

## ETHICS STATEMENT

This study was approved by our local ethics committee and written informed consent was obtained from all patients. All procedures performed in studies involving human participants were in accordance with the ethical standards of the institutional and/or national research committee and with the 1964 Helsinki Declaration and its later amendments or comparable ethical standards. This article does not contain any studies with animals performed by any of the authors.

## INFORMED CONSENT

Informed consent was obtained from all individual participants included in the study.

## Data Availability

The data that support the findings of this study are available from the corresponding author upon reasonable request.

## APPENDIX 1

STROBE Statement—Checklist of items that should be included in reports of cohort studies

**Table:**

	Item No	Recommendation	Page
Title and abstract	1	(a) Indicate the study’s design with a commonly used term in the title or the abstract	1
		(b) Provide in the abstract an informative and balanced summary of what was done and what was found	3
Introduction			
Background/rationale	2	Explain the scientific background and rationale for the investigation being reported	5
Objectives	3	State specific objectives, including any prespecified hypotheses	5
Methods			
Study design	4	Present key elements of study design early in the paper	5
Setting	5	Describe the setting, locations and relevant dates, including periods of recruitment, exposure, follow‐up and data collection	5–6
Participants	6	(a) Give the eligibility criteria, and the sources and methods of selection of participants. Describe methods of follow‐up	5–6
		(b) For matched studies, give matching criteria and number of exposed and unexposed	
Variables	7	Clearly define all outcomes, exposures, predictors, potential confounders and effect modifiers. Give diagnostic criteria, if applicable	5–7
Data sources/measurement	8*	For each variable of interest, give sources of data and details of methods of assessment (measurement). Describe comparability of assessment methods if there is more than one group	7
Bias	9	Describe any efforts to address potential sources of bias	7
Study size	10	Explain how the study size was arrived at	6
Quantitative variables	11	Explain how quantitative variables were handled in the analyses. If applicable, describe which groupings were chosen and why	7
Statistical methods	12	(a) Describe all statistical methods, including those used to control for confounding	7
		(b) Describe any methods used to examine subgroups and interactions	
		(c) Explain how missing data were addressed	
		(d) If applicable, explain how loss to follow‐up was addressed	
		(e) Describe any sensitivity analyses	
Results			
Participants	13*	(a) Report numbers of individuals at each stage of study—e.g., numbers potentially eligible, examined for eligibility, confirmed eligible, included in the study, completing follow‐up, and analysed	7–8
		(b) Give reasons for non‐participation at each stage	
		(c) Consider use of a flow diagram	
Descriptive data	14*	(a) Give characteristics of study participants (e.g., demographic, clinical, social) and information on exposures and potential confounders	7–8
		(b) Indicate number of participants with missing data for each variable of interest	
		(c) Summarize follow‐up time (e.g., average and total amount)	
Outcome data	15	Report numbers of outcome events or summary measures over time	7–8
Main results	16	(a) Give unadjusted estimates and, if applicable, confounder‐adjusted estimates and their precision (e.g., 95% confidence interval). Make clear which confounders were adjusted for and why they were included	7–8
		(b) Report category boundaries when continuous variables were categorized	
		(c) If relevant, consider translating estimates of relative risk into absolute risk for a meaningful time period	
Other analyses	17	Report other analyses done—e.g., analyses of subgroups and interactions, and sensitivity analyses	7–8
Discussion			
Key results	18	Summarize key results with reference to study objectives	9
Limitations	19	Discuss limitations of the study, taking into account sources of potential bias or imprecision. Discuss both direction and magnitude of any potential bias	10
Interpretation	20	Give a cautious overall interpretation of results considering objectives, limitations, multiplicity of analyses, results from similar studies, and other relevant evidence	9–10
Generalizability	21	Discuss the generalizability (external validity) of the study results	9–10
Other information			
Funding	22	Give the source of funding and the role of the funders for the present study and, if applicable, for the original study on which the present article is based	2

*Give information separately for exposed and unexposed groups.

*Note*: An explanation and elaboration article discusses each checklist item and gives methodological background and published examples of transparent reporting. The STROBE checklist is best used in conjunction with this article (freely available on the websites of PLoS Medicine at http://www.plosmedicine.org/, Annals of Internal Medicine at http://www.annals.org/, and Epidemiology at http://www.epidem.com/). Information on the STROBE initiative is available at http://www.strobe‐statement.org.
